# Longitudinal follow up of data‐driven cognitive subtypes in Parkinson's disease

**DOI:** 10.1002/brb3.3218

**Published:** 2023-08-13

**Authors:** Dana Pourzinal, Jihyun Yang, Kumareshan Sivakumaran, Katie L. McMahon, Leander Mitchell, John D. O'Sullivan, Gerard J. Byrne, Nadeeka N. Dissanayaka

**Affiliations:** ^1^ Faculty of Medicine The University of Queensland Centre for Clinical Research Herston QLD Australia; ^2^ School of Clinical Sciences, Faculty of Health Queensland University of Technology Brisbane QLD Australia; ^3^ School of Psychology The University of Queensland St. Lucia Australia; ^4^ Department of Neurology Royal Brisbane and Women's Hospital Herston QLD Australia; ^5^ Mental Health Service Royal Brisbane and Women's Hospital Herston QLD Australia

**Keywords:** clinical neuropsychology, cognitive impairment, mild cognitive impairment, Parkinson's disease

## Abstract

**Aim:**

The dual syndrome hypothesis proposes that there are two cognitive subtypes in Parkinson's disease (PD): a frontal subtype with executive/attention impairment and gradual cognitive decline, and a posterior‐cortical subtype with memory/visuospatial deficits and rapid cognitive decline. We aimed to compare the rate of global cognitive decline between subtypes derived using data‐driven methods and explore their longitudinal performance within specific cognitive domains to better understand the prognosis of each subtype.

**Method:**

Frontal, posterior‐cortical, globally impaired, and cognitively intact PD subtypes were identified at baseline using k‐means clustering (*N* = 85), and 29 participants (34%) returned for follow‐up assessments on average 4.87 years from baseline. Linear mixed effects models compared progression of subtypes on global cognition; psychological symptoms; parkinsonism; and the memory, attention, executive, language, and visuospatial cognitive domains.

**Results:**

The frontal subtype was lost to attrition. While rate of change in parkinsonism, anxiety, and apathy differed between subtypes, there was no difference in the rate of global cognitive decline. However, the posterior‐cortical subtype declined most rapidly in verbal memory, card sorting, trail making, and judgement of line orientation (JLO), while the cognitively intact group declined most rapidly on verbal memory and semantic fluency. The globally impaired subtype declined most rapidly in JLO, although this should be interpreted with caution due to high attrition.

**Conclusion:**

Despite limited sample size, the present study supports the differential progression of the posterior‐cortical subtype compared to cognitively intact and globally impaired PD. These results encourage further, large‐scale longitudinal investigations of cognitive subtypes in PD.

## Abstract 2

Data‐driven machine learning was used to derive cognitive subtypes in Parkinsons' disease, and their cognition was assessed at a five year follow up. Rates of decline differed across subtypes on specific domains of cognition. Results demonstrate that machine learning can identify patients with distinct cognitive trajectories over time.

## Graphical abstract

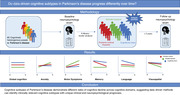

## 1 INTRODUCTION

Cognitive impairment is prevalent in Parkinson's disease (PD), although symptoms can range from mild to severe, affect any combination of cognitive domains, and progress at different rates from person to person (Aarsland et al., 2021). As a result of this heterogeneity, a vast literature on subtyping in PD has emerged (Pourzinal et al., 2022). The goal of subtyping is to create clinically homogenous subsets of patients. This allows for more precise targeting of therapies to the relevant patients; a factor on which the success of clinical trials is critically dependent (Greenland et al., 2019). Defining subtypes with shared underlying pathologies would also improve the efficiency of studies which aspire to define biological markers of the disease and its progression (Cova & Priori, 2018).

The dual syndrome hypothesis purports that there are two distinct cognitive subtypes in PD (Kehagia et al., 2012). The frontal subtype presents with attentional and executive dysfunction and gradual decline in cognition, while the posterior‐cortical subtype presents with memory and visuospatial impairment and rapid progression toward PD dementia (PDD). However, evidence of the longitudinal progression of these subtypes is limited. Previously, we used data‐driven clustering methodology to identify the dual syndrome subtypes in a sample of 85 people with PD without dementia (Pourzinal et al., 2020). Cognitively intact and globally impaired subtypes were also revealed, which reflected cognitively “normal” participants and participants showing frontal *and* posterior‐cortical impairments, respectively.

The present study aimed to evaluate the predictions of the dual syndrome hypothesis and explore prognoses of the data‐driven cognitive subtypes from our original study (Pourzinal et al., 2020). The primary outcome was the rate of change in global cognitive function, which was assessed longitudinally and compared across subtypes. Secondary outcomes such as change in parkinsonism, psychological, and domain‐specific cognitive measures were also explored. In line with the dual syndrome hypothesis, it was predicted that the posterior‐cortical subtype would exhibit more rapid global cognitive decline over time compared to the frontal subtype. Given the extent of cognitive impairment of the globally impaired subtype at baseline, it was predicted that this group would exhibit the fastest decline over time. Conversely, the cognitively intact subtype was predicted to remain cognitively intact over time.

## 2 METHODOLOGY

### 2.1 Participants

All 85 participants from the original study were invited to participate in the present study (Pourzinal et al., 2020). This cohort was recruited mostly from movement disorders outpatient clinics in Brisbane, Australia, with some recruited from the community. Only participants who had received deep brain stimulation (DBS) were excluded due to its potential effect on cognition (Bucur & Papagno, 2022). Baseline assessments took place between 2016 and 2018 and follow‐up assessments were completed in 2021–2022. All participants were assessed in the “ON” state while on their levodopa medications. All participants provided informed, written consent prior to participation in the study, and the protocol received approval from the Royal Brisbane and Women's Hospital (HREC/10/QRBW/285) and University of Queensland (UQ2010001297) Human Research Ethics Committees.

### 2.2 Clustering

The clustering methodology used to delineate cognitive subtypes in this sample has been described elsewhere (Pourzinal et al., 2020). Briefly, 10 baseline cognitive measures were categorized into “frontal” and “posterior‐cortical” cluster variables based on previous literature, and data available from all participants were entered into several k‐means cluster analyses. The cluster structure of best fit identified four groups of patients with unique patterns of cognitive impairment across the frontal and posterior measures: (1) globally impaired; (2) posterior‐cortical impaired; (3) frontal impaired; and (4) cognitively intact.

### 2.3 Measures

Self‐report measures included the Geriatric Depression Scale‐15 (GDS) (Weintraub et al., 2006), Parkinson's Anxiety Scale (PAS) (Leentjens et al., 2014), and Starkstein Apathy Scale (SAS) (Starkstein et al., 1992) to assess depression, anxiety, and apathy, respectively. Parkinsonism and global cognition were measured using the Movement Disorder Society Unified Parkinson's Disease Rating Scale (MDS‐UPDRS) (Goetz et al., 2008) and Montreal Cognitive Assessment (MoCA) (Nasreddine et al., 2005), respectively. Levodopa equivalent daily dose (LEDD) was calculated using the method cited in Tomlinson et al. (2010). Participants were assessed at level II of the MDS guidelines for identifying Mild Cognitive Impairment in PD (PD‐MCI) using a battery of 10 cognitive tests from five domains (Litvan et al., 2012). Attention was measured with the Trail Making Test A (TMT‐A) (Lezak, 2012) and the color‐word scale of the STROOP (Golden & Freshwater, 2002) test. Executive function was measured using Trail Making Test B (TMT‐B) (Lezak, 2012) and card sorting from the Delis–Kaplan Executive Function System (D‐KEFS) (Delis et al., 2004). Memory was measured with Hopkins Verbal Learning Test‐Revised (HVLT) and Brief Visuospatial Memory Test‐Revised (BVMT) delayed recall scales (Benedict & Brandt, 2007). Visuospatial function was measured using Benton's judgement of line orientation (JLO) (Calamia et al., 2011) and CLOX (Royall et al., 1998) clock drawing task. Finally, language was assessed with the D‐KEFS (Delis et al., 2004) semantic fluency task and Boston Naming Test (BNT) (Kaplan et al., 1983). To meet PD‐MCI criteria, participants needed to score at least 1.5*SD* below age‐ and, where available, education‐adjusted normative scores on at least two measures. Alternative forms were used where possible (MoCA, D‐KEFS, BVMT, HVLT, and JLO) in the follow‐up assessment to mitigate practice effects.

### 2.4 Statistics

Baseline demographic data was compared using Mann–Whitney *U* tests for continuous variables and *χ* ^2^ goodness of fit tests for categorical variables, at a significance threshold of *p* < .05. Linear mixed‐effects models were created to compare the rate of change in outcome measures between the groups (Molenberghs & Verbeke, 2001). In all models, cognitive subtype was the independent variable and age was a covariate, both entered as fixed variables in the model. In the models with cognitive outcomes, education was added as an additional covariate. An interaction term was included between time and subtype group, and participant ID was included as a random variable in all models. To evaluate change in global cognition over time, MoCA was used as the primary outcome measure and the significance threshold was set to Bonferroni‐adjusted *p* < .05. To explore change in psychological, parkinsonism, and domain‐specific cognitive measures, GDS, PAS, SAS, MDS‐UPDRS, and each cognitive measure of the level 2 PD‐MCI test battery were outcome measures in separate models. Due to the exploratory nature of the analyses, the significance threshold for these secondary outcome measures was set to non‐adjusted *p* < .05. Supporting Information 1 details the full parameters for each model reported in the results. Categorical clinical variables were compared using Phi and Cramer's V. Continuous clinical variables were compared using one‐way ANOVA where parametric assumptions were met, Kruskal–Wallis tests where assumptions were not met, and Bonferroni‐corrected post hoc tests at an adjusted significance threshold of *p* < .05. All statistical comparisons were performed using SPSS 27.0.1.0 (Armonk, NY). Linear mixed effects modeling and visualization were performed using R version 3.6.3 and R studio version 1.4.1717. Models were created using the *lmerTest* package, (Kuznetsova et al., 2017) and visualized using the *dplyr* and *ggplot2* packages (dplyr: A Grammar of Data Manipulation, 2023; ggplot2: Elegant Graphics for Data Analysis. New York, 2016).

## 3 RESULTS

### 3.1 Participants

**FIGURE 1:**
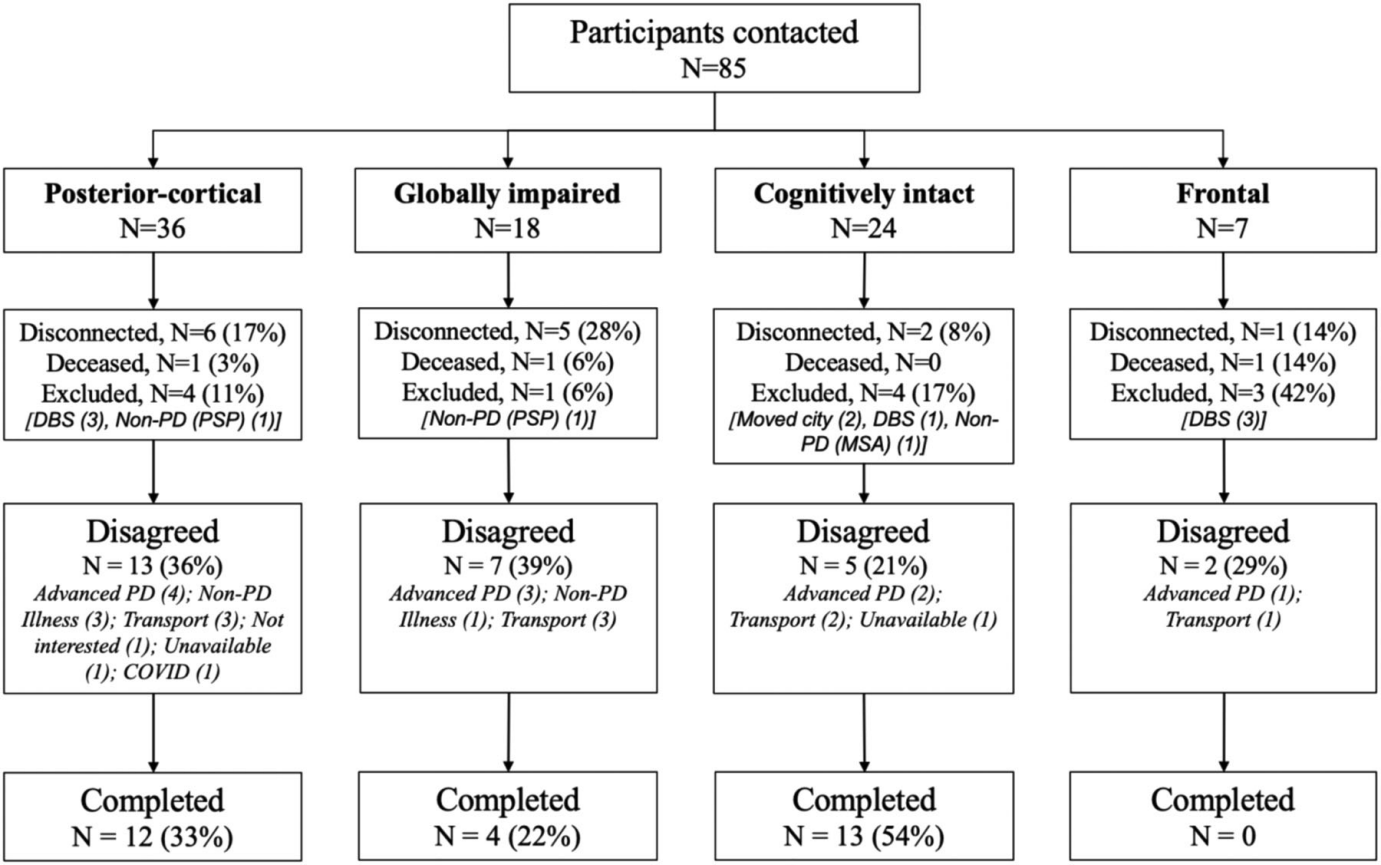
Recruitment flowchart indicating reasons for attrition.

Of the 85 participants from the original study, 29 completed the study; 7 were excluded due to DBS, 3 had passed away, 3 were no longer diagnosed with PD, 10 declined due to advanced PD, 15 declined for other reasons, and 18 were lost to follow up. No participants were taking acetylcholinesterase inhibitors at baseline or follow up. Figure 1 displays the recruitment breakdown for each subtype. In total, 12 participants from the cognitively intact group, 12 patients from the posterior‐cortical group, and 4 patients from the globally impaired group completed the study. The highest rate of exclusion due to DBS was in the frontal subtype (42%), whereas the globally impaired subtype had the highest overall attrition rate and the highest rate of drop out due to illness. Due to lack of participants, the frontal subtype was unable to be included in statistical analyses. Baseline and follow‐up demographic information are summarized for each subtype in Table 1.

**TABLE 1:** Baseline and follow‐up sample characteristics, Mean *(SD)* / *N* (%).

	Subtype			
Clinical variable	Posterior‐cortical (*N* = 12)	Globally impaired (*N* = 4)	Cognitively intact (*N* = 13)	All PD (*N* = 29)
**Age** (years)				
Baseline	67.83 (7.82)	71.75 (2.63)	62.15 (6.75)	65.57 (7.60)
Follow up	72.17 (7.94)	76 (1.41)	66.31 (6.46)	70.07 (7.5)
**Sex** (*female*)	4 (33%)	1 (25%)	9 (69%)	
**Education** (years)				
Baseline	10.56 (3.32)	10.25 (3.30)	14.38 (2.60)	12.37 (3.47)
Follow up	11.83 (4.57)	11.50 (4.44)	15.23 (3.65)	13.37 (4.3)
**Dis. duration** (years)				
Baseline	5.41 (3.73)	2.5 (1.73)	3.62 (2.21)	4.20 (3.33)
Follow up	10.25 (3.57)	7 (3.56)	8.23 (2.09)	8.9 (3.12)
**Follow‐up duration** (years)	5.00 (.69)	4.52 (1.20)	4.81 (0.77)	4.88 (0.79)
**LEDD**				
Baseline	449.57 (213.76)	366.67 (57.7)	465.29 (390.89)	428.95 (295.16)
Follow up	884.98 (406.89)	898.44 (408.39)	724.32 (441.52)	818.05 (415.03)
**MDS‐UPDRS‐Total**				
Baseline	32.70 (15.32)	37.25 (14.13)	31.07 (13.76)	32.54 (13.75)
Follow up	57.00 (28.00)	69.5 (17.06)	46.25 (21.95)	54.18 (24.78)
**MDS‐UPDRS‐Motor**				
Baseline	16.75 (7.88)	25.25 (10.08)	14.54 (7.80)	16.77 (8.55)
Follow up	27.83 (15.40)	46.25 (10.14)	22.58 (11.85)	28.21 (15.09)
**MoCA**				
Baseline	25.41 (2.78)	23.50 (4.79)	27.00 (1.73)	25.87 (2.82)
Follow up	25.08 (2.19)	23.75 (3.95)	28.38 (1.39)	26.38 (2.82)
**MCI**				
Baseline	1 (8%)	2 (50%)	0 (0%)	3 (10%)
Follow up	9 (75%)	3 (75%)	3 (21%)	15 (50%)

Abbreviations: Dis., disease; GDS, Geriatric Depression Scale; LEDD, levodopa equivalent daily dose; MCI, mild cognitive impairment; MDS‐UPDRS, Movement Disorders Society Unified Parkinson's Disease Rating Scale; MoCA, Montreal Cognitive Assessment; PAS, Parkinson's Anxiety Scale.

### 3.2 Missing data analysis

Table 2 summarizes comparisons of baseline demographic variables between those who returned to complete the study and those who did not. The only significant difference between completers and non‐completers was evident in MCI status, with a greater proportion of MCI among non‐completers compared to completers. Those with MCI at baseline were also more likely to drop out of the study (OR = 4.5; 95%CI = 1.17, 17.25). The data was therefore likely missing at random (Little & Rubin, 2019), as it is plausible that non‐completers did not attend the follow up due to their greater cognitive impairment. Figure 1 provides evidence for this, with PD‐related illness (including cognitive impairment) being the most frequent reason for non‐participation. Missing data were treated using Available Case Analysis and the implications of the bias introduced using this method is addressed in the discussion (Jakobsen et al., 2017).

**TABLE 2:** Comparison of baseline demographic variables between completers and non‐completers.

	Completers (*N* = 29)		Non‐completers (*N* = 53)			
Clinical variable	*Mean / N* (%)	*SD*	*Mean / N* (%)	*SD*	Test statistic	*p*‐value
Age (years)	65.83	7.57	69.74	8.70	*U* = 567	**.050**
Sex (*female*)	14 (48%)		20 (38%)		*χ* ^2^ = 0.035	.853
Education (years)	12.43	3.32	13.20	3.57	*U* = 575	.293
Disease duration (years)	3.76	3.00	5.50	6.77	*U* = 646	.287
LEDD	454.52	292.85	476.44	289.56	*U* = 715	.607
MDS‐UPDRS total	32.59	14.01	41.94	22.85	*U* = 553	.098
MDS‐UPDRS motor	16.93	8.60	21.91	13.57	*U* = 624	.161
MoCA	25.86	2.88	24.81	2.70	*U* = 571	.053
GDS	3.79	3.52	3.83	3.03	*U* = 686	.673
PAS	10.66	7.24	9.08	7.06	*U* = 659	.350
MCI	3 (11%)		17 (32%)		*χ* ^2^ = 9.80	**.002**

*Note*: Bolded values indicate statistical significance.

Abbreviations: GDS, Geriatric Depression Scale; LEDD, levodopa equivalent daily dose; MCI, mild cognitive impairment; MDS‐UPDRS, Movement Disorders Society Unified Parkinson's Disease Rating Scale; MoCA, Montreal Cognitive Assessment; PAS, Parkinson's Anxiety Scale.

### 3.3 Global cognitive decline

**FIGURE 2:**
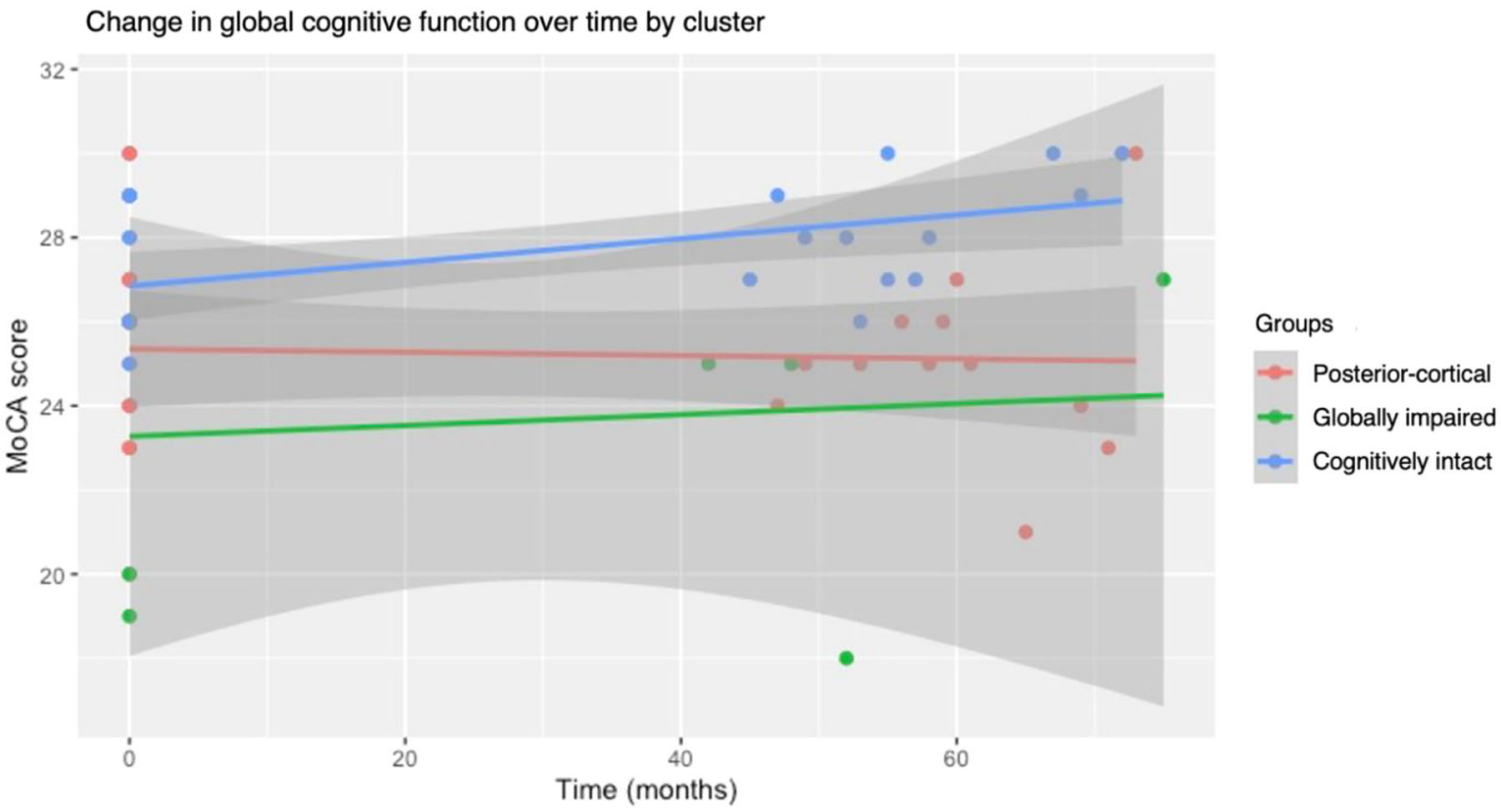
Change in global cognition over time by subtype.

The resulting linear mixed effects model was: moca ∼ GROUP * Time + Age + Education + (1 | Participant number). Figure 2 visually depicts the change in global cognition for each subtype over time. Only the intercept was significant (*β* = 28.92, 95%CI = 21.59–36.26, *p* < .001), indicating that baseline MoCA scores differed between groups. However, the interaction between time and the posterior‐cortical (*β* = 0, 95%CI = −0.03 to 0.02, *p* = .722), globally impaired (*β* = 0.02, 95%CI = −0.03 to 0.07, *p* = .496), and cognitively intact (*β* = 0.03, 95%CI = −0.00 to 0.06, *p* = .068) subtypes were all non‐significant, indicating no difference in the rate of global cognitive decline between groups.

### 3.4 Parkinsonism and psychological symptoms

**FIGURE 3:**
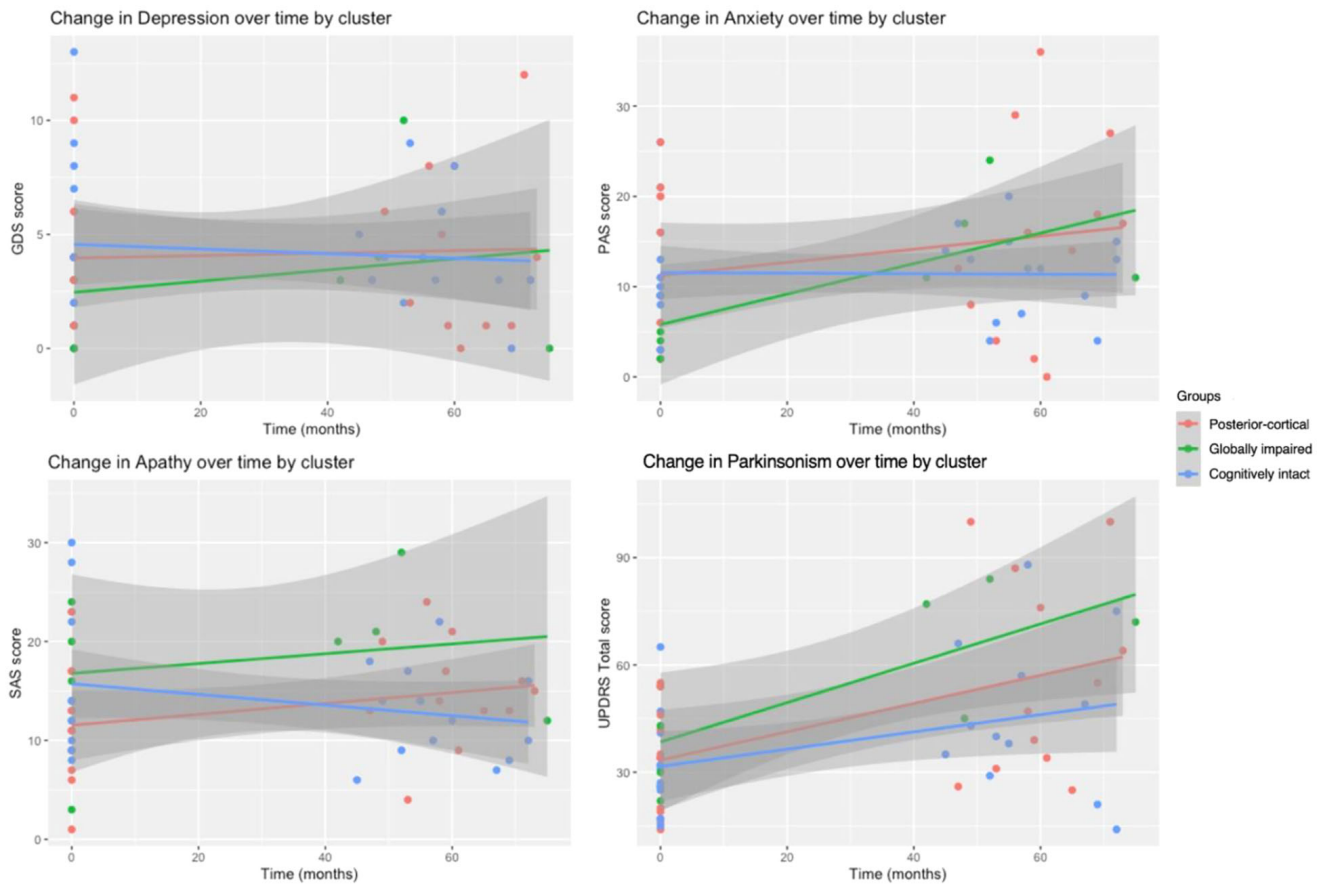
Change in depression, anxiety, apathy and parkinsonism measures over time by subtype. GDS, Geriatric Depression Scale; PAS, Parkinson's Anxiety Scale; SAS, Starkstein Apathy Scale; UPDRS, Movement Disorders Society Unified Parkinson's Disease Rating Scale.

The model parameters for the psychological and parkinsonism measures are provided in Table 3. Figure 3 visually depicts the change in each measure for each subtype over time. There was no difference in the rate of change of depression over time between subtypes. The interaction between anxiety and time was significant for the posterior‐cortical and globally impaired groups, indicating rapid increase for the globally impaired, moderate increase for the posterior‐cortical and no increase for the cognitively intact subtype. Only the cognitively intact subtype significantly differed in the rate of change in apathy; where the other two subtypes showed an increase, the cognitively intact group decreased over time. Finally, the posterior‐cortical group differed in the rate of change in parkinsonism such that their UPDRS score increased more rapidly than the cognitively intact subtype yet less rapidly than the globally impaired subtype.

**TABLE 3:** Linear mixed effect model parameters for psychological symptoms and parkinsonism.

	Model							
	Intercept		Posterior‐cortical × Time		Globally impaired × Time		Cognitively intact × Time	
Test	*β*	*p*	*β*	*p*	*β*	*p*	*β*	*p*
GDS	12.78	**.023**	0.00	.864	0.03	.358	−0.01	.600
PAS	13.15	.338	0.06	**.031**	0.12	**.050**	−0.07	.102
SAS	19.32	.087	0.04	.073	0.04	.408	−0.10	**.004**
MDS‐UPDRS	56.96	.124	0.40	**<.001**	0.19	.221	−0.16	.134

*Note*: Bolded values indicate statistical significance.

Abbreviations: GDS, Geriatric Depression Scale; MDS‐UPDRS, Movement Disorders Society Unified Parkinson's Disease Rating Scale; PAS, Parkinson's Anxiety Scale; SAS, Starkstein Apathy Scale.

### 3.5 PD‐MCI cognitive test battery

The model parameters for each cognitive measure are provided in Table 4. Subtypes significantly differed at baseline (intercept) on each cognitive measure except the two TMT scales. Interaction results are discussed by cognitive domain later.

**TABLE 4:** Linear mixed effect model parameters for cognitive measures of the PD‐MCI battery.

		Model							
		Intercept		Posterior‐cortical × Time		Globally impaired × Time		Cognitively intact × Time	
Domain	Test	*β*	*p*	*β*	*p*	*β*	*p*	*β*	*p*
Memory	HVLT delayed	11.49	**<.001**	−0.05	**<.001**	0.06	**.006**	0.03	**.048**
	BVMT delayed	7.87	**.042**	0.01	.559	−0.02	.596	−0.01	.709
Language	CF	60.26	**<.001**	−0.09	**.003**	0.07	.279	−0.13	**.003**
	BNT	64.57	**<.001**	−0.01	.224	0.01	.713	0.00	.922
Attention	TMT‐A	−27.64	.208	0.27	**<.001**	0.01	.972	−0.20	**.034**
	STROOP	29.14	**.034**	−0.08	**.022**	−0.01	.868	0.05	.274
Executive function	TMT‐B	−81.31	.277	1.32	**<.001**	0.11	.833	−1.05	**.002**
	Card sorting	73.74	**<.001**	−0.21	**<.001**	−0.03	.740	0.07	.230
Visuospatial function	CLOX	28.19	**<.001**	−0.02	.119	−0.00	.866	0.01	.488
	JLO	23.74	**<.001**	−0.05	**.004**	−0.08	**.039**	0.02	.501

*Note*: Bolded values indicate statistical significance.

Abbreviations: BVMT, Brief Visuospatial Memory Test‐Revised; BNT, Boston Naming Test; HVLT, Hopkins Verbal Learning Test‐Revised; JLO, Benton's judgement of line orientation; TMT, Trail Making Test.

#### 3.5.1 Memory

**FIGURE 4:**
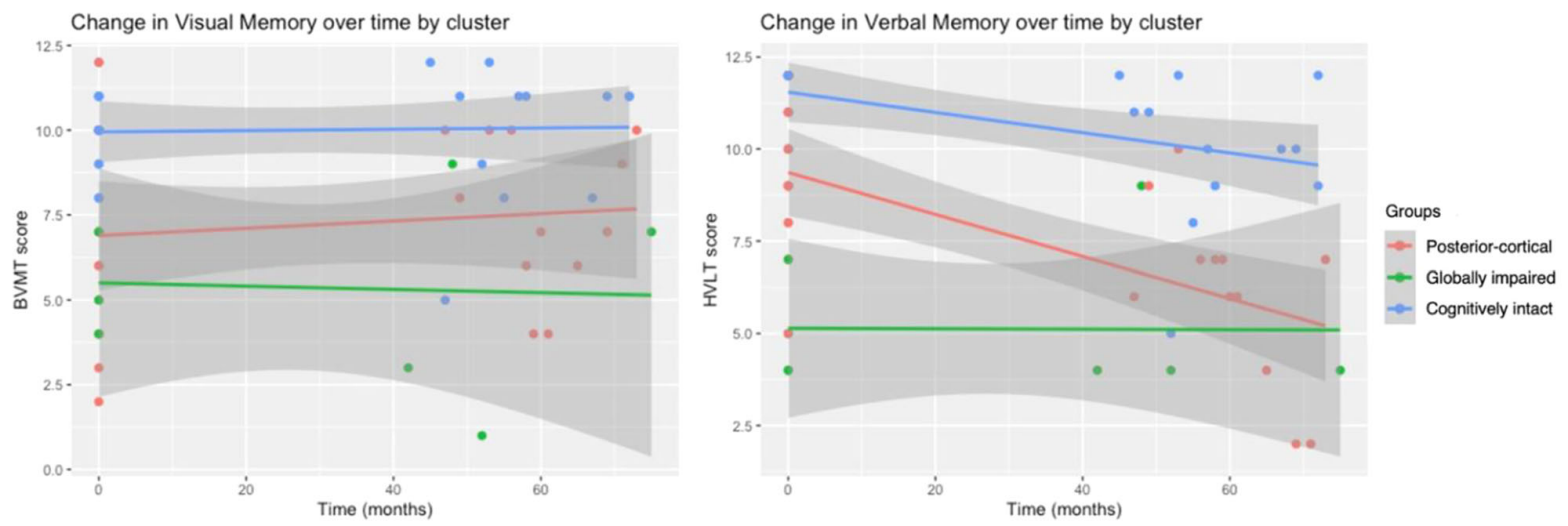
Change in memory domain measures over time by subtype.

Figure 4 visually depicts change in memory for each subtype over time. Subtypes did not differ in their rate of change on visual memory. Change in verbal memory over time was significantly different, with the posterior‐cortical group exhibiting the greatest decline, followed by the cognitively intact. The globally impaired group performed consistently poorly over time.

#### 3.5.2 Language

**FIGURE 5:**
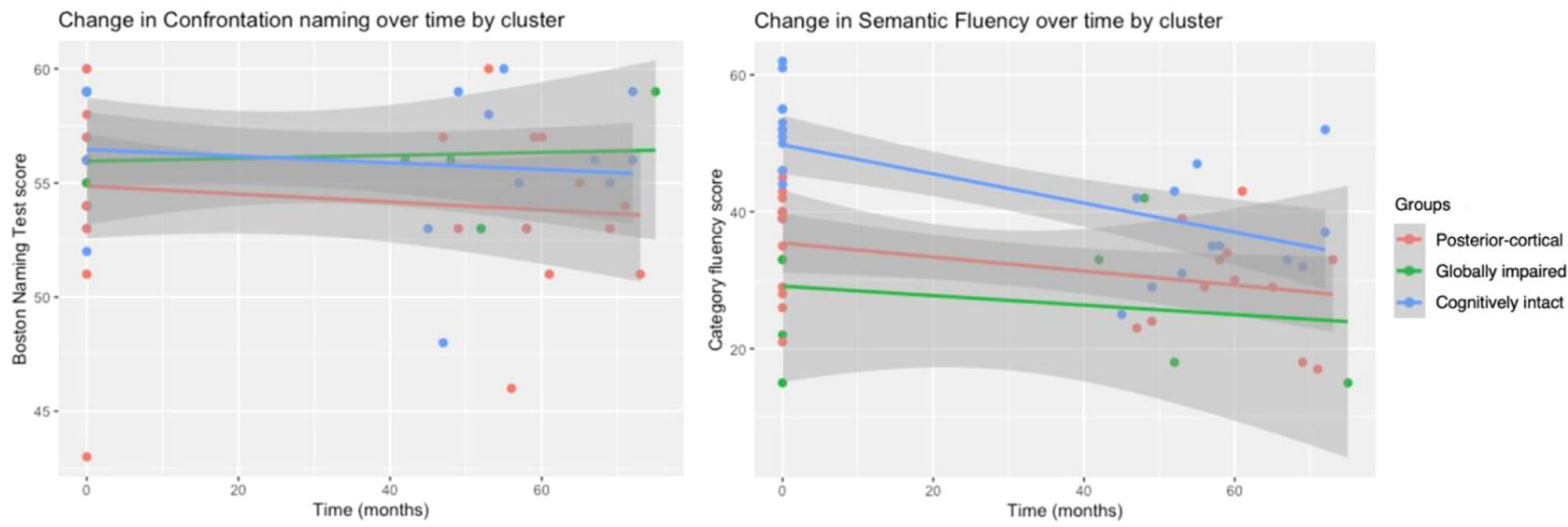
Change in language domain measures over time by subtype.

Figure 5 visually depicts change in language ability for each subtype over time. Subtypes demonstrated similar rates of change on confrontation naming (BNT). However, significant differences in rate of change over time were revealed for semantic fluency, where the cognitively intact subtype exhibited the greatest decline followed by the posterior‐cortical subtype.

#### 3.5.3 Attention

**FIGURE 6:**
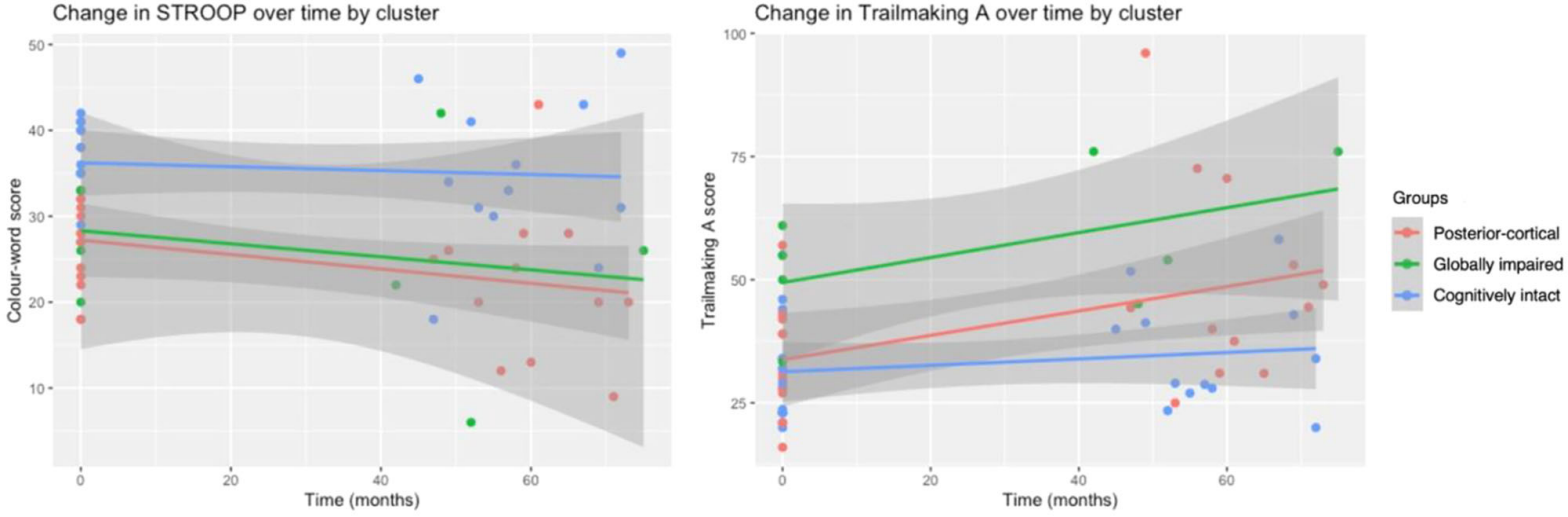
Change in attention domain measures over time by subtype.

Figure 6 visually depicts change in attention for each subtype over time. Significant differences in rate of change over time were revealed for both the STROOP and TMT‐A measures. The posterior‐cortical and globally impaired subtypes exhibited similarly rapid decline, whereas the cognitively intact group remained relatively stable.

#### 3.5.4 Executive function

**FIGURE 7:**
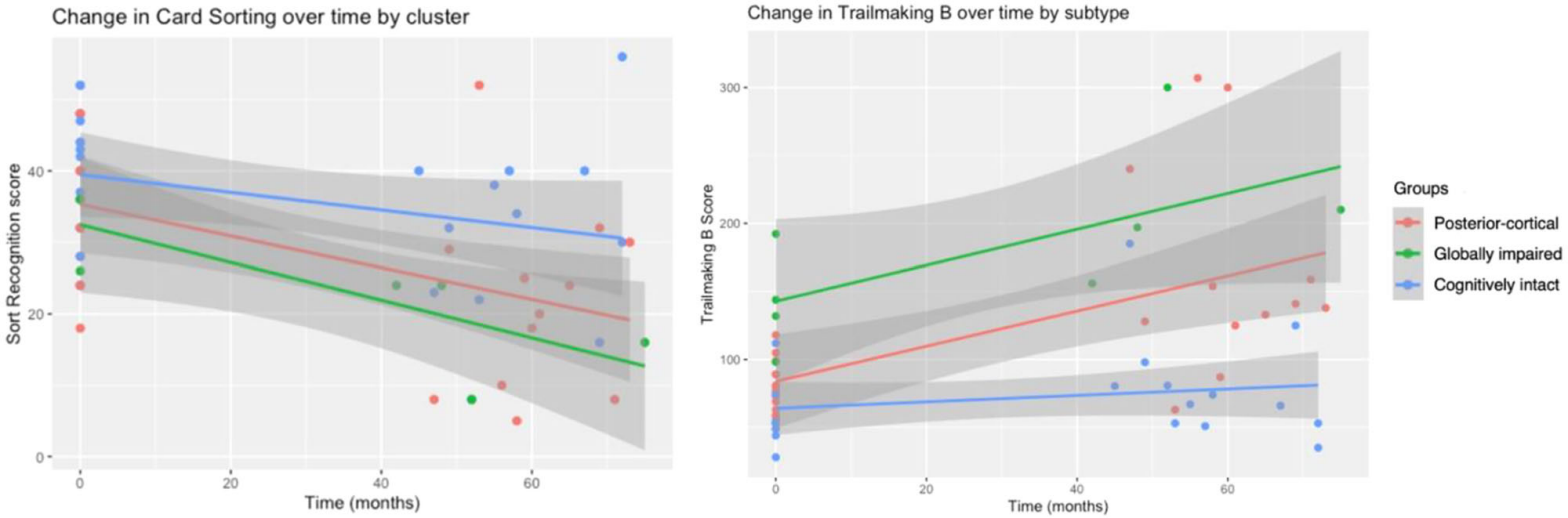
Change in executive domain measures over time by subtype.

Figure 7 visually depicts change in executive function for each subtype over time. Significant differences in rate of change over time were revealed for both the card sorting and TMT‐B measures. While all groups showed some decline on card sorting, the posterior‐cortical and globally impaired subtypes exhibited greater decline. Similarly, the posterior‐cortical and globally impaired subtypes demonstrated rapid decline on TMT‐B whereas the cognitively stable group remained relatively stable.

#### 3.5.5 Visuospatial function

**FIGURE 8:**
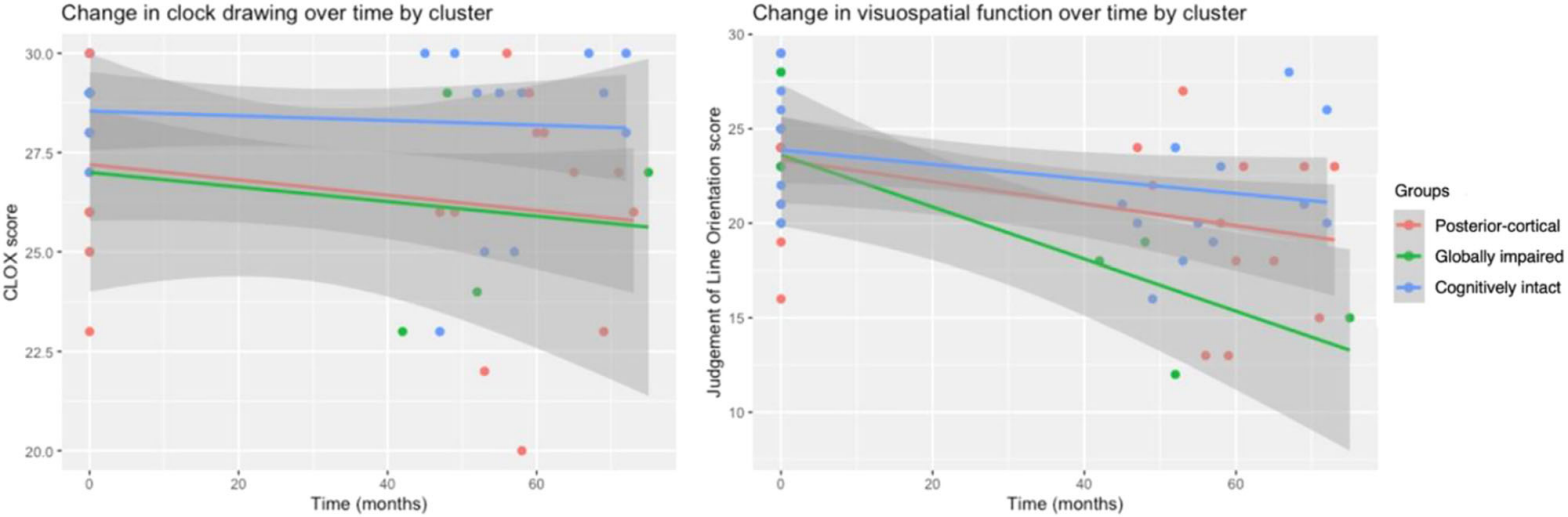
Change in visuospatial domain measures over time by subtype.

Figure 8 visually depicts change in visuospatial function for each subtype over time. Rate of change in clock drawing did not differ across groups. However, significant differences in the rate of change of JLO were revealed. The globally impaired group showed the greatest decline, followed by the posterior‐cortical subtype, while the cognitively intact subtype showed only some decline over time.

## 4 DISCUSSION

The present study is the first to follow the cognitive trajectories of data‐driven subtypes in PD. Unfortunately, the frontal subtype was excluded due to attrition and thus the primary aim of comparing the frontal and posterior‐cortical subtypes could not be addressed. The findings instead revealed critical differences in the progression of cognitive symptoms across the cognitively intact, posterior‐cortical, and globally impaired subtypes. However, these subtypes also suffered from high attrition rates and results should therefore be interpreted with caution as an exploratory investigation.

### 4.1 Summary of results

Contrary to our predictions, no differences in the rate of change in global cognitive function were revealed across subtypes. Notably, the overall mean MoCA scores in Table 1 appeared to show an upward trend with higher scores at follow up compared to baseline. This contradicts the reported prevalence of MCI in the sample, which increased over time. Although alternative forms were used, the upward trend may be explained by practice effects to which the MoCA was shown to be susceptible (Cooley et al., 2015). Nevertheless, assuming that practice effects were equal among groups, differences in the rate of change in global cognition would still be expected. This suggests that participants who returned to complete the study may have had little cognitive change. However, it is also possible that the MoCA simply was not sensitive to decline in specific cognitive domains and thus did not reflect the changes that occurred in each subtype accurately.

Change in psychological and parkinsonism measures also differed across subtypes. While depression remained stable for the globally impaired and posterior‐cortical groups, anxiety increased. Given the positive relationship between anxiety and cognitive impairment (Dissanayaka et al., 2017), these findings align with the literature. Depression and anxiety remained stable for the cognitively intact group, although levels of apathy waned relative to the increase of the globally impaired and posterior‐cortical groups. It is probable that this increase is related to the increased age, cognitive impairment, and parkinsonism of these groups, which are all known risk factors for apathy (den Brok et al., 2015). Parkinsonism increased steadily over time for all groups, with the greatest increase for the globally impaired followed by the posterior‐cortical and then cognitively intact groups. This finding aligns with the literature reporting a positive association between cognitive impairment and motor symptom severity in PD (Aarsland et al., 2021).

While all subtypes generally declined in performance on the PD‐MCI battery, exploratory analyses revealed several differences in terms of decline in specific cognitive domains. The posterior‐cortical group only remained stable in visual memory and declined most rapidly in verbal memory, card sorting, trail making, and JLO. Since visuospatial and memory dysfunction is characteristic of the posterior‐cortical syndrome, the observed changes are mostly unsurprising. However, decline in card sorting and trail making is perhaps a reflection of increasing impairments becoming more pervasive and impacting adjacent cognitive domains. Notably, the synchronous decline in verbal memory and increase in anxiety aligns with the literature linking verbal memory deficits to anxiety in PD (Dissanayaka et al., 2022).

Interestingly, the cognitively intact and globally impaired subtypes appeared to show inverse trends. The cognitively intact group exhibited relatively stable attention, executive, and visuospatial function but rapid decline in verbal memory and semantic fluency. While this decline likely does not reflect clinically significant impairment given the low proportion of MCI cases and high MoCA scores in this subtype, it does suggest perhaps a newfound risk of rapid cognitive decline as verbal memory and semantic fluency are well‐known predictors (Compta et al., 2013; Domellöf et al., 2015; Hobson & Meara, 2015). Conversely, the globally impaired group maintained poor but stable memory and language function while declining in attention, executive, and visuospatial function, with the greatest decline in JLO. The high proportion of MCI cases and low MoCA scores suggests this is the result of pervasive cognitive decline in this subtype, as expected.

### 4.2 What can attrition tell us?

Due to attrition, change in cognition could not be quantitatively evaluated for the frontal subtype. However, insight into each subtype's disease progression can be gained from exploring reasons for attrition. For example, the frontal subtype reported the highest proportion of DBS treatment since baseline (Figure 1). This may indicate greater motor symptom complexity in this group as severe motor fluctuations and dyskinesia are critical criteria for DBS selection (Dallapiazza et al., 2018), raising questions about the relationship between motor symptomatology and frontal dysfunction in PD. Furthermore, the posterior‐cortical and globally impaired subtypes had the highest rate of drop out due to advanced PD. This corroborates the findings of the study, which showed more rapid decline in parkinsonism and cognition in these groups compared to the cognitively intact.

### 4.3 Implications

The present study failed to evaluate the predictions of the dual syndrome hypothesis. However, the findings show the utility of machine learning to identify people with PD at risk of cognitive decline. Of the posterior‐cortical participants who completed the study, only one had PD‐MCI at baseline (Pourzinal et al., 2020). Yet, this subtype progressed more rapidly than the cognitively intact in terms of cognitive decline, psychological symptoms, and parkinsonism. This suggests that identification of the dual syndrome subtypes through cluster analysis was successful at capturing those at risk of rapid cognitive decline above and beyond the conventional PD‐MCI criteria.

Furthermore, the results highlight specific cognitive tests and their role in measuring cognitive decline in PD. For example, the rate of decline in JLO was markedly different across groups despite similar baseline scores. In contrast, group differences in visual memory were distinct at baseline even though they did not decline over time. These finding suggest that changes in visual memory may occur earlier in the disease and plateau, whereas visuospatial dysfunction may occur later in the disease. Additionally, the greatest decline of all measures occurred for the posterior‐cortical group on verbal memory and globally impaired group on JLO. These measures should therefore be prioritized for these patient groups as indicators of cognitive decline. While the MoCA is an excellent clinical tool, it did not reflect the specific cognitive changes revealed in the comprehensive PD‐MCI test battery. For this reason, while it may be a practical screening tool to identify global cognitive impairment in the clinic, the MoCA is not recommended for those seeking to explore the heterogeneity of cognitive symptoms in PD.

### 4.4 Strengths and limitations

The obvious limitation of the study is its lack of sample size due to high attrition. Selective attrition due to advanced PD and cognitive impairment also meant that those remaining in the study reflected those with milder PD and cognitive symptoms. This issue is pertinent to cognitive research in older adults (Salthouse, 2019), and results in a systematic underestimation of the rate of cognitive decline with greater bias for groups with higher attrition. Thus, the rates of decline for the posterior‐cortical and globally impaired subtypes are likely much greater in reality. Furthermore, the severe disease and cognitive characteristics of the globally impaired subtype at baseline may confound the findings, with differences in cognitive progression of this group potentially reflecting differences in cognitive stage as opposed to cognitive subtype. Results for this group should be interpreted with caution. Regardless, the results provide qualitative insights into the progression of each subtype and a rudimentary evaluation of cognitive decline in PD, highlighting important differences in the progression of specific cognitive symptoms.

Another limitation is the lack of a healthy control sample, without which it is impossible to infer whether the cognitive decline experienced by the cognitively intact PD subtype is comparable with “normal ageing.” Decreasing the follow‐up intervals (e.g., 18 months) would also provide more nuanced information about change in cognition over time. Finally, it is important to acknowledge the intricate interconnectivity of the brain and subsequent overlap of “frontal” and “posterior” networks, as well as the inherent overlap across the five cognitive domains (i.e., memory, executive, visuospatial, language, and attention). While no domain can work independently, these distinctions are nevertheless important to make in research to identify cognitive subtypes and their trajectory over time.

## 5 CONCLUSIONS

Although the rate of change in global cognitive function did not differ between groups, the present study revealed unique differences in longitudinal cognitive decline between data‐driven cognitive subtypes in PD. The posterior‐cortical subtype declined most rapidly in verbal memory, card sorting, trail making, and JLO, indicative of proliferating cognitive decline across multiple domains. The globally impaired subtype exhibited widespread declines in executive, attention, and visuospatial measures, whereas the cognitively intact group demonstrated the least overall cognitive decline and incidence of MCI over time, with the greatest decline in semantic fluency and verbal memory. The dual syndrome hypothesis has proven valuable in identifying cognitive subtypes in PD with discrete disease progression, although further longitudinal investigation with larger samples is needed to verify the results.

## AUTHOR CONTRIBUTIONS

**Dana Pourzinal**: Conceptualization (equal); funding acquisition (lead); investigation ‐ data collection (equal); writing ‐ original draft (lead); formal analysis (lead); writing ‐ review and editing (equal). **Jihyun Yang**: Supervision (equal); investigation ‐ data collection (equal); Review and editing (equal). **Kumareshan Sivakumaran**: Investigation ‐ data collection (equal); writing ‐ review and editing (equal). **Katie Mitchell**: Supervision (equal); writing ‐ review and editing (equal). **John O'Sullivan**: Supervision (equal); writing ‐ review and editing (equal). **Gerard Bryne**: Supervision (equal); writing ‐ review and editing (equal). **Nadeeka Dissanayaka**: Supervision (equal); conceptualization (equal); funding acquisition (supporting); Writing ‐ review and editing (equal).

## CONFLICT OF INTEREST STATEMENT

The authors declare no conflicts of interests.

### PEER REVIEW

The peer review history for this article is available at https://publons.com/publon/10.1002/brb3.3218

## Data Availability

Data may be available upon request.
